# Treatment outcomes of implant-assisted removable partial denture with distal extension based on the Kennedy classification and attachment type: a systematic review

**DOI:** 10.1186/s40729-021-00394-z

**Published:** 2021-11-13

**Authors:** Adityakrisna Yoshi Putra Wigianto, Takaharu Goto, Yuki Iwawaki, Yuichi Ishida, Megumi Watanabe, Tetsuo Ichikawa

**Affiliations:** Department of Prosthodontics & Oral Rehabilitation, Tokushima University Graduate School of Biomedical Sciences, 3-18-15 Kuramoto, Tokushima, 770-8504 Japan

**Keywords:** Dental implant, Implant-assisted removable partial denture, Kennedy classification, Attachment, Treatment outcomes

## Abstract

**Background:**

Implant-assisted removable partial dentures (IARPDs) have recently become popular, but little information is available on the treatment outcomes based on the Kennedy classification and attachment types.

**Objective:**

The objective of this review was to evaluate the treatment outcomes of IARPD delivered for distal extension edentulous areas based on the differences in the Kennedy classification and attachment type.

**Materials and methods:**

English-language clinical studies on IARPD published between January 1980 and February 2020 were collected from MEDLINE (via PubMed), the Cochrane Library (via the Cochrane Central Register of Controlled Trials), Scopus online database, and manual searching. Two reviewers selected the articles based on pre-determined inclusion and exclusion criteria, followed by data extraction and analysis.

**Results:**

Eighty-one studies were selected after evaluating the titles and abstracts of 2410 papers. Nineteen studies were finally included after the perusal of the full text. Fourteen studies focused on Class I, 4 studies investigated both Class I and II, and only 1 study was conducted on Kennedy’s class II. Eight types of attachments were reported. The ball attachment was the most frequently used attachment, which was employed in 8 of the included studies. The implant survival rate ranged from 91 to 100%. The reported marginal bone loss ranged from 0.3 mm to 2.30 mm. The patient satisfaction was higher with IARPD than with conventional RPDs or that before treatment. The results of prosthetic complications were heterogeneous and inconclusive.

**Conclusion:**

IARPD exhibited favorable clinical outcomes when used as a replacement for distal extension edentulous areas. The comparison between the clinical outcomes of Kennedy’s class I and II was inconclusive owing to the lack of studies focusing on Kennedy Class II alone. The stud attachment was the most commonly used type in IARPDs. Overall, the different attachment systems did not influence the implant survival rate and patient satisfaction. Further high-quality studies are needed to investigate the attachment systems used in IARPD.

## Introduction

At the outset, osseointegrated implants were used to support bone anchored-bridges in patients with complete edentulism, and the application of implant-based prostheses was gradually extended to partial edentulism. Currently, implant-based prostheses can be used for the rehabilitation of all types of edentulous spans with predictable outcomes.

Dental implants have also been used widely to support overdentures, leading to extensive evaluations for decades. The McGill consensus statement on overdentures published in 2002 stated that mandibular two-implant overdentures are the first choice of treatment for completely edentulous patients [1]. This treatment modality improves denture retention and stability with the help of a few osseointegrated implants, which also helps to limit the treatment cost [2]. Subsequently, the application of dental implants to support overdentures was expanded to removable partial dentures (RPD). Several current epidemiological studies have reported a decrease in the frequency of complete edentulism, which is expected to exhibit a declining trend in future years, owing to the increase in the availability of dental healthcare facilities. Thus, the number of patients requiring RPD treatment has increased [3, 4]. The frequency for RPD treatment is the highest for Kennedy’s class I, followed by Kennedy’s class II [5]. However, the prosthodontic replacement of these types of edentulous arches is beset by several challenges such as unfavorable movements of the RPD due to the differences between the viscoelasticity of the oral mucosa and abutment teeth, retention loss, mucosal irritation or ulceration, and discomfort arising from the retentive clasps [6, 7]. Implant-assisted RPDs (IARPDs) are a viable option that can overcome the above-mentioned issues and limitations of conventional RPDs (CRPDs) [7]. The use of an implant to assist the RPD confers some advantages, such as improved retention, stability, patient comfort, patient satisfaction, confidence, reduction of denture movement under the fulcrum line, decreased requirement for relining, and reduced risk of combination syndrome [8, 9]. Several studies have investigated the clinical outcomes and viability of IARPD. Several review studies have also been published on this topic. De Freitas RF et al. reported on the patient satisfaction, survival rate of implants, and prosthetic complications of mandibular IARPDs in 2012 [10]. Subsequently, numerous studies have reported on this aspect, including various case reports. A systematic review and meta-analysis conducted by Park et al. evaluated the treatment outcome after replacing the CRPD with IARPD in patients with Kennedy’s class I in the mandibular arch [11]. However, from the clinical perspective, Kennedy’s class II is as important as Kennedy’s class I.

Moreover, various types of attachment systems are used in IARPDs. The selection of these attachments is usually based on several considerations, such as the amount of retention needed, inter-arch space, patient dexterity, and the clinician’s skill [12]. Aldhohrah et al. conducted a systematic review and meta-analysis on different attachment systems used in mandibular implant overdentures (IOD) [13]. The available attachment types used in IARPD are similar to those used in IOD, but the biomechanical conditions of the two prostheses are not the same. However, no study has investigated the different types of attachment used against the prosthesis survival rates and other clinical parameters in patients with Kennedy’s Class I and II treated with IARPD.

This systematic review was conducted to evaluate the treatment outcomes of IARPD with distal extension based on the differences in the Kennedy classification and attachment type, in addition to a comprehensive evaluation of the latest findings on this topic.

## Materials and methods

This systematic review was conducted according to the Preferred Reporting Items for Systematic Reviews and Meta-analysis (PRISMA) [14] with following the PICO (*P* = patient problem/population, *I* = intervention, *C* = comparison, *O* = outcomes) model:

- Population: patients with Kennedy classification I or II either on maxilla or mandibula
- Intervention: Implant Assisted Removable Partial Dentures
- Comparison: Kennedy classification and attachment system
- Outcome: clinical outcomes, such as implant survival rate, marginal bone loss, patient satisfaction.

### Information sources and search

The English language literature published between January 1980 and February 2020 were extracted using the MEDLINE (via PubMed), Cochrane Library (via Cochrane Central Register of Controlled Trials, CENTRAL), and Scopus databases. The electronic database search was performed using keywords and MeSH terms based on the following search strategy used for exploring MEDLINE (via PubMed): (("Denture, Partial"[Mesh]) AND ("Dental Prosthesis, Implant-Supported"[Mesh] OR "Dental Implants"[Mesh])) OR (implant-assisted removable partial denture [Title/Abstract]). A manual search was also performed in addition to these database searches by checking the bibliography of all identified articles for potentially relevant additional studies.

### Inclusion criteria

The studies were selected based on the following inclusion criteria: (1) studies with patients with Kennedy’s class I or II (treated with distal extension RPD); (2) case reports, cohort studies, or randomized controlled trials (RCTs); (3) studies that reported clinical outcomes, such as marginal bone loss or survival rate of implants, periodontal conditions of the abutment teeth, or patient satisfaction; (4) studies in which the attachment system was clearly described; and (5) studies whose full-text was available in English.

### Exclusion criteria

The following studies were excluded: in vitro studies, animal studies, and review studies, studies that did not mention the method of measuring clinical outcomes, and those reporting qualitative outcomes descriptions without presenting the exact values.

### Study selection

**Fig. 1:**
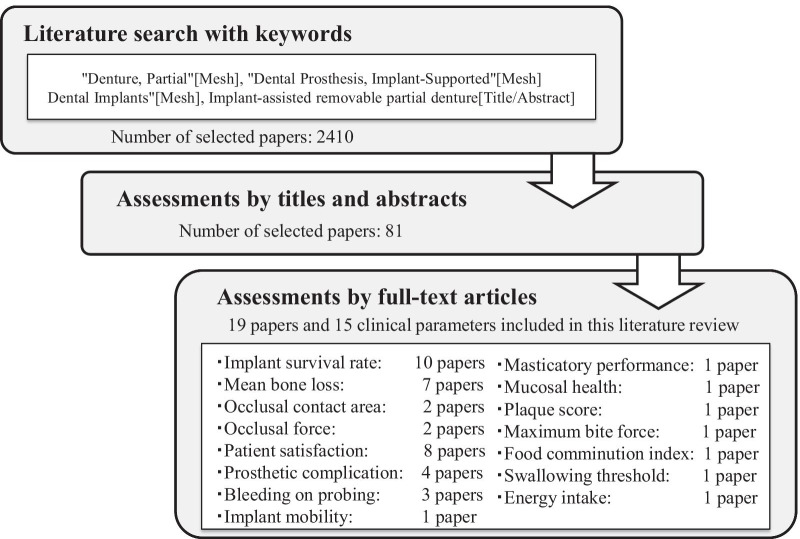
Literature review strategy

Figure 1 demonstrates the literature search strategy used in this study. Two authors (T.G. and A.Y.P.W.) who had previously determined the criteria independently evaluated the literature search. First, the collected titles and abstracts were selected according to the aim and pre-determined criteria. Second, two reviewers confirmed the concurrence of the results, and the full-text of these articles was read to further examine the details of the results reported. Subsequently, the discrepancies in the results of the two authors were discussed with a third reviewer (T.I.). Finally, the studies that investigated the prognosis and IARPD outcomes with respect to the attachment systems were included.

### Data collection process and data items

An extraction sheet was created using Microsoft Excel (Microsoft Office Professional Plus 2019, CA, USA) for data collection. The table contained the following information: author, publication year, research design, follow-up period, number of patients, number of implants, attachment design, and results. The literature review was performed after summarizing the results based on each subfield.

## Results

### Study selection

A total of 2410 studies published between 1986 and 2020 were obtained according to the search strategy described in Fig. 1. Eighty-one studies were selected for full-text assessment after initial screening of the titles and abstracts. Nineteen studies were finally selected after the application of the inclusion criteria. Based on the year of publication, the earliest included study was published in 2003[15]. Three studies were from the 2000s [15–17], and 16 studies were from the 2010s and 2020 [18–33]. Three RCTs had the highest level of evidence with respect to the study design [25–27], followed by 2 randomized crossover trials [16, 31], 9 prospective studies [18–24, 30, 33], 2 retrospective studies [15, 28], and 3 case reports [17, 25, 29].

The current review focused on two Kennedy classifications, class I and II. Most of the studies were based only on class I [16, 19, 20, 22–28, 30–33], followed by studies that included both class I and II cases (4 studies) [15, 18, 21, 29], and only one study investigated class II cases[17]. The treatment outcomes were not described separately or compared between these 2 classes in the four studies that included both classes.

Eight different types of denture attachments were utilized in the included studies. The ball attachment was the most frequently used attachment (8) [19, 20, 22–24, 28, 30, 33], followed by the locator (5) [17, 18, 26, 27, 29], healing abutment [15, 16], equator [32], stress breaking ball [31], ball with clix [21], extra-coronal resilient attachment (ERA) [25], and resilient attachment [15]. However, the treatment outcomes between those different attachments were compared only in one study [15]. Fifteen clinical parameters were extracted from the included studies.

This review also attempted to include published IARPD studies that dealt with the maxillary and mandibular arches. Nevertheless, we did not find a single study that evaluated the use of IARPD in the maxillary arch only. A majority of the included studies dealt only with the mandibular arch [15–17, 19–22, 24–28, 30–33]; meanwhile, only 3 studies included both maxillary and mandibular arches [18, 23, 29].

### Clinical outcomes

**Fig. 2:**
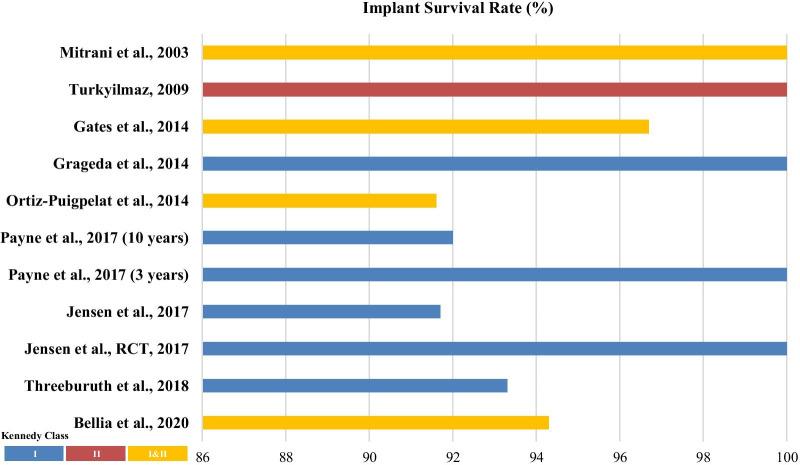
Comparison of the implant survival rate in the included studies based on Kennedy’s classification of the study samples [*Payne et al. reported the survival rates for 3 years and 10 years, separately [30]]

**Fig. 3:**
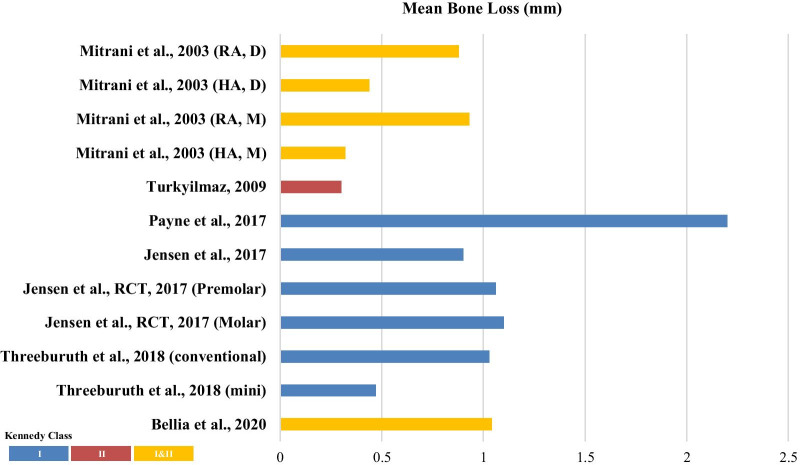
Comparison of the mean bone loss between the included studies based on Kennedy classification of the study populations. *RA* resilient attachment, *HA* healing abutment, *D* distal surface, *M* mesial surface, *Conventional* conventional sized implant, *Mini* mini implants

The characteristics of the selected studies are summarized in Table 1. The treatment outcomes of studies that investigated only Kennedy’s class I or class II were implant survival rate and marginal bone loss. The comparison of these two clinical outcomes based on Kennedy’s classification is presented in Figs. 2 and 3.

**Table.1:** Treatment outcomes of IARPD reported by the included studies

Implant survival rate								
Author	Year	Research design	Follow-up period	Number of patients	Number of implants	Kennedy classification	Attachment design	Results
Bellia E, et al.	2020	Prospective	4 years	Total: 20 U: –, L: –	U: 7, L: 28	I&II	Locator	94.3%
Threeburuth W, et al.	2018	RCT	12 months	L: 30	L: 60	I	Equator on mini implant	93.3%
Jensen C, et al.	2017	RCT	3 months	L: 30	L: 120	I	Locator	100%
Jensen C, et al.	2017	Retrospective	3–16 years Mean: 8 years	L: 23	L: 46	I	Ball	91.7%
Payne AG, et al.	2017	Prospective	3 and 10 years	L: 36	L: 72	I	Ball	Survival after 3 years: 100% Survival after 10 years: 92%
Ortiz-Puigpelat O, et al.	2014	Case report	Mean: 28.6 months	U: 6, L: 6	U: 12, L: 12	I&II	Locator	91.6%
Grageda E, et al.	2014	Case report	3 years	L: 1	L: 2	I	ERA	100%
Gates WD 3^rd^, et al.	2014	Prospective	6 and 12 weeks	L: 17	L: 30	I&II	Ball with Clix	96.7%
Turkyilmaz I	2009	Case Report	18 months	L: 1	L: 2	II	Locator	100%
Mitrani R, et al.	2003	Retrospective	1 to 4.5 years Mean: 2.52 years	U: 4, L: 6	U: 5, L: 6	I&II	Healing abutment, resilient attachment	100%

Mean bone loss								
Bellia E, et al.	2020	Prospective	4 years	Total: 20 U: –, L: –	U: 7, L: 28	I&II	Locator	1.04 ± 1.88 mm
Threeburuth W, et al.	2018	RCT	12 months	L: 30	L: 60	I	Equator on mini implant	Mini implant: 0.47 ± 0.42 mm, Conventional implant: 1.03 ± 1.07 mm Mini vs. conventional implant*
Jensen C, et al.	2017	RCT	3 months	L: 30	L: 120	I	Locator	Molar (M) Implant: 1.10 ± 0.53, Premolar (PM) Implant: 1.06 ± 0.59 *M vs. PM*^*(−)*^
Jensen C, et al.	2017	Retrospective	3–16 years Mean: 8 years	L: 23	L: 46	I	Ball	Mean: 0.9 ± 1.0 mm; Anterior: 1.0 ± 1.1 mm; Posterior: 0.8 ± 1.0 mm *Anterior vs. posterior *^*(−)*^
Payne AG, et al.	2017	Prospective	3 and 10 years	L: 36	L: 72	I	Ball	2.20 ± 0.81 mm
Turkyilmaz I	2009	Case report	18 months	L: 1	L: 2	II	Locator	0.3 ± 0.1 mm
Mitrani R, et al.	2003	Retrospective	1–4.5 years Mean: 2.52 years	U: 4, L: 6	U: 5, L: 11	I&II	Healing abutment, Resilient attachment	Mesial (total: 0.61 ± 0.62): *Healing abutment (0.32* ± *0.47) vs. Resilient attachment (0.93* ± *0.64) *^*(−)*^ Distal (total: 0.64 ± 0.45): *Healing abutment (0.44* ± *0.45) vs. Resilient attachment (0.88* ± *0.34) *^*(−)*^

Occlusal contact area								
Suzuki Y, et al.	2017	Randomized crossover	2–3 weeks	L: 10	L: 20	I	Stress Breaking Ball (SBB)	CRPD: around 4 mm^2^, Healing abutment: around 7.8 mm^2^, SBB: around 7.8 mm^2^ CRPD vs. healing abutment & SBB*
Ohkubo C, et al.	2008	Single-blinded randomized crossover	2–3 weeks	L: 5	L: 10	I	Healing abutment	ISRPD on RPD area: around 6 mm^2^, CRPD on RPD area: around 3 mm^2^, ISRPD on full dental arch: 10 mm^2^, CRPD on full dental arch: 8 mm^2^ ISRPD vs. CRPD*

Occlusal force								
Suzuki Y, et al.	2017	Randomized crossover	2–3 weeks	L: 10	L: 20	I	Stress Breaking Ball (SBB)	Conventional RPD: around 200 N, Healing abutment: around 400 N SBB: 400 N CRPD vs. healing abutment & SBB*
Ohkubo C, et al.	2008	Crossover	2–3 weeks	L: 5	L: 10	I	Healing abutment	ISRPD on RPD area: around 300 N, CRPD on RPD area: around 50 N ISRPD on full dental arch: around 550 N, CRPD on full dental arch: around 400 N ISRPD vs. CRPD* (ISRPD had significant greater force)

Patient satisfaction								
Threeburuth W, et al.	2018	RCT	12 months	L: 30	L: 60	I	Equator on mini implant	Comfort, Retention, Chewing performance Before vs. after treatment*; mini-implant vs. conventional implant^*(−)*^
Jensen C, et al.	2016	RCT	3 and 6 months	L: 30	L: 120	I	Locator	OHIP/total (Max score: 196): Old RPD = 49.6, New RPD = 40.3, ISRPD(M) 17.6, ISRPD(PM) = 21.2 ISRPD vs. RPD*
Campos CH, et al.	2015	Prospective	2 months	L: 12	L: 24	I	Ball	QoL assessment using OHIP indicators & other indicators, such as chewing difficulty, pronunciation, appearance, self-consciousness ISRPD with Ball abutment vs. CRPD*
Ortiz-Puigpelat O, et al.	2014	Case Report	Mean: 28.6 months	U; 6, L: 6	U: 12, L: 12	I&II	Locator	VAS Score Before = 1.19 ± 0.64, After = 4.55 ± 0.35 IARPD vs. RPD*
Goncalves TM, et al.	2014	Prospective	2 months	Total: 12 U: -, L: -	Total: 24 U: -, L: -	I	Ball	VAS Score on retention, comfort, masticatory capacity, speaking ability ISRPD vs. CRPD*
Wismeijer D, et al.	2013	Prospective	3 years	L; 48	L: 72	I	Ball	OHIP, OHIQ, VAS IARPD vs. CRPD*
Ohkubo C, et al.	2008	Crossover	2–3 weeks	L: 5	L: 10	I	Healing abutment	VAS Score on stability, chewing, retention, and comfort ISRPD vs. CRPD*
Mitrani R, et al..	2003	Retrospective	1–4.5 years Mean: 2.52 years	U: 4, L: 6	U: 5, L: 11	I&II	Healing abutment, Resilient attachment	Questionnaire (1–5 scale, 1 was least favorable): before (1.2), after (5.0) Before vs. after IARPD Treatment*

Technical/prosthetic complication								
Jensen C, et al.	2017	Retrospective	3–16 years Mean: 8 years	L: 23	L: 46	I	Ball	No complications: 4 Anterior(A), 11 Posterior(P) = 15 (65.21%) Minor repair: 2 (1A, 1P) (8.68%), Replaced: 3 (2A, 1P) (13%), Not in function: 1 (A) (4.34%) Reverted into a full arch denture: 2 (P) (8.68%) Anterior vs. Posterior* (anterior implant had significantly higher complications)
Ortiz-Puigpelat O, et al.	2014	Case Report	Mean: 28.6 months	U; 6, L: 6	U: 12, L: 12	I&II	Locator	Attachment (Locator) loosening: 0 (0%), Retentive cap mobility: 6 (50%) Plastic retentive male change: all patients at 12th-month visit (100%) Denture teeth wear: 7 (1 requires change) (58.33%), Metal framework: 1 broken after 15 months (8.33%)
Gates WD 3^rd^, et al.	2014	Prospective	6 and 12 weeks	L: 17	L: 30	I&II	Ball with Clix	Clasp adjustments: 5 (29.41%), Denture base relining: 2 (11.76%), Fracture on denture tooth: 2 (11.76%), Abutment loosening: 1 (5.88%), Attachments replacement: 1 (5.88%), RPD reprocessing: 1 (5.88%)

Bleeding on probing								
Bellia E, et al.	2020	Prospective	4 years	Total: 20 U: –, L: –	U: 7, L: 28	I&II	Locator	BOP rate at 1 year: 20%, BOP rate at 4 years: 36.36%
Jensen C, et al.	2017	RCT	3 months	L: 30	L: 120	I	Locator	Molar support: 0.26 ± 0.29, Premolar support: 0.11 ± 0.11 Molar vs. Premolar*
Jensen C, et al.	2017	Retrospective	3–16 years Mean: 8 years	L: 23	L: 46	I	Ball	Anterior: 0.5(0.6), Posterior: 0.8(0.6) *Anterior vs. Posterior*^*(−)*^

Implant mobility								
Bellia E, et al.	2020	Prospective	4 years	Total: 20 U: –, L: –	U: 7, L: 28	I&II	Locator	Mobility rate on 1 year: 0%, Mobility rate on 4 years: 3.03%

Masticatory performance								
Goncalves TM, et al.	2014	Prospective	2 months	L: 12	L: 25	I	Ball	VAS Score RPD vs. IRPD & IFPD*

Mucosal health								
Jensen C, et al.	2017	Retrospective	3–16 years Mean: 8 years	L: 23	L: 46	I	Ball	Gingiva Index: Anterior: 0.1 ± 0.3, Posterior: 1.1 ± 0.7, Anterior vs. Posterior* Probing depth: Anterior: 3.3(1.4); Posterior: 3.3(1.2), A*nterior vs. Posterior*^*(−)*^

Plaque score								
Jensen C, et al.	2017	Retrospective	3–16 years Mean: 8 years	L: 23	L: 46	I	Ball	Plaque Index (total: 0.9 ± 0.7): Anterior: 0.6 ± 0.7; Posterior: 1.1 ± 0.7 Anterior vs. Posterior*

Maximum bite force								
Goncalves TM, et al.	2013	Clinical trial	2 months	L: 12	L: 48	I	Ball	RDP: 178.1 ± 36.4 N*,* IRDP: 318.4 ± 47.1 N, IFDP: 483.9 ± 50.5 N RDP vs. IRDP* (140 N gain, 79%), RDP vs. IFDP* (306 N gain, 172%)

Food comminution index								
Goncalves TM, et al.	2013	Clinical trial	2 months	L: 12	L: 48	I	Ball	RDP: 19.9 ± 5.3%, IRDP: 38 ± 7.7%*,* IFDP: 61.6 ± 10.6% RDP vs. IRDP* (91% increase), RDP vs. IFDP* (209% increase)

Swallowing threshold								
Campos CH, et al.	2014	Prospective	2 months	L: 8	L: 16	I	Ball	Masticatory cycles don’t differ significantly between prosthesis treatments X50: CRPD = 3.64; IARPD = 2.92 IARPD vs. CRPD*

Energy intake								
Campos CH, et al.	2014	Prospective	2 months	L: 8	L: 16	I	Ball	Parameters: Energy (kcal), Carbohydrate (g/day), Protein (g/day), Calcium (mg/day), Fiber (mg/day), Iron (mg/day) ISRPD vs. IARPD*

*RPD* Removable partial denture, *IARPD* Implant-assisted removable partial denture, *ISRPD* Implant-supported removable partial denture, *CRPD* Conventional removable partial denture, *IFPD* Implant fixed partial denture, *IRPD* Implant-retained partial denture, *ERA* Extracoronal resilient attachment, *RCT* Randomized controlled trial. *OHIP* Oral health impact profile, *OHIQ* Oral health impact questionnaire, *VAS* Visual analog scale, *STTI* Swallowing threshold test index, *significant difference, ^(−)^no significant difference

### Implant survival rates

Overall, the implant survival rates reported by the included studies ranged from 91% to 100%. Jensen et al*.* reported a 100% implant survival rate after 3 months of evaluation [26]. Similar survival rates (100%) were also reported by Grageda et al*.* [25], Turkyilmaz [17], and Mitrani et al*.* [15] after a mean evaluation period of 3 years, 18 months, and 2.52 years, respectively. Payne et al*.* incorporated the longest evaluation period (10 years), which yielded a 92% survival rate [30]. The lowest survival rate (91.6%) was reported by Ortiz-Puigpelat et al*.*, who evaluated in 12 patients for a mean duration of 28.6 months [29]. All studies reported high implant survival rates, irrespective of the attachment type. The implant survival rates of the included studies (Fig. 2) based on the Kennedy classification were as follows: class I, 91.7–100%; class II, 100%; and classes I and II, 91.6–100%.

### Marginal bone loss

Eight studies included in this review reported the mean bone loss data, with the reported mean value ranging from 0.3 mm to 2.30 mm. Only one study by Threeburuth et al*.* compared the use of mini-implants with conventional implants and reported significantly lower marginal bone loss with the mini-implant with equator attachment group [33]. Mitrani et al*.* compared the healing outcomes between the healing abutment and resilient attachments [15]. However, other studies did not report any significant differences in the outcomes with respect to the different implant types, positions, or attachment systems. The highest marginal bone loss was reported by Payne et al*.*, which was as high as 2.20 ± 0.81 mm [30]. The different types of attachments did not specifically increase or decrease the marginal bone loss. Figure 3 depicts the comparison between the different Kennedy classes (mean values, class I: 0.47–2.20 mm, class II: 0.3 mm, and classes I and II: 0.32–1.04 mm).

### Patient satisfaction

Eight of the 19 studies included in this review reported patient satisfaction outcomes using precise values and pre-determined parameters. Six studies, which were conducted by Jensen et al*.* [27], Campos et al*.* [19], Ortiz-Puigpelat et al*.* [29], Goncalves et al*.* [23], Wismeijer et al*.* [33], and Ohkubo et al*.* [16] compared the differences between the implant-supported RPDs (ISRPD)/IARPD and CRPD. All studies stated that patients reported significantly better satisfaction with ISRPD/IARPD over CRPD. Threeburuth et al*.* [32] and Mitrani et al*.* [15] compared this outcome between before and after ISRPD/IARPD treatment, and both studies found a significant increase in patient satisfaction after treatment. However, Threeburuth et al*.* found no significant difference between the use of mini-implants and conventional implants [32]. IARPD treatment increased patient satisfaction compared to that before treatment or CRPD usage, irrespective of the attachment types and Kennedy’s classification.

### Technical/prosthetic complications

Three of the included studies reported the prosthetic/technical complication outcomes for IARPDs [21, 28, 29]. Jensen et al*.* reported that 15 of 23 prostheses used ball attachments did not have complications [28]. Ortiz-Puigpelat et al*.* did not find any locator abutment loosening; however, all plastic retentive components (matrix) had to be changed after 12 months. Some other complications were also observed [29]. Gates et al*.* used a ball with a clip attachment and reported that one prosthesis needed attachment replacement, one RPD needed reprocessing and so on. [21]. Thus, the described technical/prosthetic complication data were very heterogeneous, since each study utilized different types of attachments. A more detailed description of studies reporting technical/prosthetic complications is provided in Table 1.

### Other clinical outcomes

Other clinical outcomes, such as the occlusal contact area, occlusal force, bleeding on probing, implant mobility, masticatory performance, mucosal health, plaque score, maximum masticatory force, food comminution index, swallowing threshold, and energy intake were also reported. Jensen et al. reported significantly higher bleeding on probing rates with implants placed in the molar region compared to the premolar region [26]. Suzuki et al*.* [31] and Ohkubo et al*.* [16] found a significantly higher occlusal force and occlusal contact area with ISRPD/IARPD compared to CRPD. Goncalves et al*.* reported that IARPD exhibited a significantly higher masticatory performance, maximal masticatory force, and food comminution index than those for CRPD [22]. Jensen et al*.* reported significantly better gingival and plaque indices for more anteriorly placed implants compared to those placed posteriorly [28]. Campos et al*.* stated that IARPD yielded a significantly better result with respect to the swallowing threshold and energy intake assessment compared to CRPD [20].

## Discussion

The terminology used by previous studies on ISRPD/IARPD lacked uniformity, even though they described similar oral conditions and prosthetic designs. We opine that the terminology for implant-based RPDs should depend on the nature of the implants’ function (support, retention, or bracing). Healing abutments only provide support in implant-supported dentures, without providing retention. On the other hand, attachments perform both functions. We considered the term “implant-assisted removable partial dentures” to be the most suitable, since our review focused on the differences between the attachment systems.

The treatment outcomes of IARPD can be compared with other treatment modalities, such as implant-supported fixed dental prostheses (ISFDP) and IOD. Pjetursson et al. reported that the implant survival rate of ISFDP was 95.6% (94.4–96.6%) at 5 years and 93.1% (90.5–95.0%) at 10 years, although the success rate of prostheses without complications was only 66.4% over 5 years [34]. The implant survival rates of IOD were 73–100% in the maxilla [35], and 71–100% for the maxillary and mandibular arches [36]. Our review found that the implant survival rate of IARPD ranged between 91.6 and 100% for the maxillary and mandibular arches. A comparison revealed that the survival rate of IARPD was acceptable, when compared to that of IOD and ISFDP, although it was not proven statistically. The disparity in the study design and evaluation period did not permit the performance of a meta-analysis in this study.

A systematic review and meta-analysis conducted by Borges et al. revealed that the ISFDP and IOD differed only with respect to the oral health-related quality of life and satisfaction, although the ISFDP tended to show comparatively better results [37]. However, other indicators, such as implant survival rate, marginal bone loss, and periodontal diseases did not show that ISFDP was more efficient than IOD. This observation could be attributed to the greater stability of fixed prostheses, which was probably responsible for better patient satisfaction [9]. Nevertheless, ISFDP is not always the best treatment choice for all patients, especially considering economic factors, availability of inter-occlusal space, remaining bone volume, and maintenance of implants and prostheses. To overcome those circumstances, IARPD can be chosen. The results of this study indicated significantly greater patient satisfaction with IARPD compared to that with CRPD or before IARPD treatment, which confirms the findings of previous reviews [10, 11, 38].

This distribution of the included studies in this review that analyzed the clinical outcomes based on the Kennedy classification was as follows: 14 studies included class I cases [16, 19, 20, 22–28, 30–33], 4 studies included class I and II cases [15, 18, 21, 29], and only one study focused on class II cases alone [17]. However, we could not compare the data of studies that incorporated a mix of class I and II cases, since the results did not clearly differentiate between the data for class I and class II in these studies. Two outcomes were compared (Figs. 2 and 3), which illustrate that the implant survival rate of IARPD for Kennedy’s class I (91.6–100%) was lower than that for Kennedy’s class II (100%). However, only a brief comparison was possible owing to the disparity in the sample size and study design between the two groups. The class II group also presented with a slightly lower level of mean bone loss (0.3 mm) than that in the class I group (0.47–2.20 mm). The nature of edentulism in Kennedy’s class I, in which fewer remaining teeth are available to provide retention and support, may result in greater instability of the CRPD and the transmission of higher lateral stress to the implants. Moreover, Resnik stated that Kennedy’s class I patients who have a higher risk of biological or prosthodontic complications need more implant support compared to most class II or III patients [39]. Biomechanically, the purpose of placing the implant with IARPD in Kennedy’s class I and II conditions is to convert an unstable, distal extension edentulous area that lacks support to a class III configuration with greater support and retention, reduces the torque on the abutment tooth, and minimizes the need for clasps in the RPD design [9, 40].

The attachment system is among the factors that can influence the outcomes of IARPD, since different attachment systems possess different characteristics and mechanisms. Most of the included studies utilized stud-type attachments (18 of 19 studies, 94.7%). The remaining study [16] used a rounded healing abutment as support. Based on the retentive mechanism, the attachment system was classified into the stud (O-ring, extra-coronal resilient attachment, ball, locator, and magnet), bar, and telescopic attachments. The stud attachment is further subclassified into the resilient and non-resilient types based on its function [12]. The ball attachment was found to be the most used attachment in the included studies, since this type of attachment is simple, cost effective, and less technique-sensitive [41].

Most clinical studies reported favorable results, despite various differences in the attachments. According to Aldhohrah et al., the implant survival rate was high both on immediate and delayed loading, irrespective of the attachment type [13]. Data on patient satisfaction outcomes from all included studies was significantly better with IARPD use, irrespective of the utilization of the stud or healing abutment in the attachment system. This result emphasizes the findings of Kim et al.’s systematic review, i.e., patient satisfaction might be independent of the attachment system used [42]. Previous reviews also showed that IARPD increased patient satisfaction despite various attachments [10, 11, 38]. Furthermore, Goto et al. investigated the effect of attachment installation conditions on the load transfer and denture movements of IOD for three types of attachments. The attachment system and its method of installation both affected the load distribution between the implants and mucosa [43].

Mitrani et al. compared the outcomes between different types of attachment (healing abutment and resilient attachment) in their retrospective study but found no significant difference between these groups after measurement on the mesial or distal side [15]. Chen et al. mentioned that the height of the implant abutment, which differs for each attachment type, influences early bone loss around the implants [44]. Each of the three studies [21, 28, 29] that evaluated the technical or prosthetic complication-related outcomes used different type of attachments, and the reported complications were too diverse and inconclusive. The most recent systematic review and meta-analysis on attachment systems in IOD were also unable to arrive at a definite conclusion due to the heterogeneity of the reported outcomes. The attachment system used in a prosthesis may influence prosthetic maintenance and complications [42]. Therefore, further investigations on this aspect are recommended.

## Study limitations

Although the overall results of the included studies showed favorable clinical outcomes, we could not analyze them statistically owing to the high degree of heterogeneity in the design, method, number of patients, and evaluation period. Regarding the evaluation period, Pandolfi et al. mentioned that peri-implant complications tend to occur after 5 years post-loading condition [45]. However, among 19 included studies, there was a follow-up for more than 5 years in only 2 studies [28, 30]. For evidence-based treatment using IARPD, further reports with long-term follow-up should be accumulated.

Ideally, case reports should not be involved in a systematic review. However, there is a lack of published studies that fit the purpose of this study. Moreover, the reported clinical outcomes are also diverse. Goodacre et al. (2003) had comprehensively reviewed complications of implant-supported prostheses and included case reports [46]. Considering the lack of evidence regarding treatment outcomes based on the attachment system and Kennedy classification in distal extension IARPD, we conducted a comprehensive review and included those three case reports in our study.

Unfortunately, we could not perform a meta-analysis due to the lack of studies with a high quality of evidence. Among the 19 included studies, there were only 3 RCTs as the highest evidence level. Those studies also have various properties in terms of patient numbers, only evaluated in a short period (3, 6, 12 months) which is insufficient for outcomes. Moreover, there is a probability of patient allocation bias in clinical studies. At this point, a more homogenous or standardized protocol for further high-quality RCTs is recommended to facilitate the comparison of the clinical outcomes based on the different kinds of attachments. In addition to attachment systems, other variables, such as implant size (mini, conventional), and implant position which also contribute to IARPD success rate should be investigated further.

## Conclusion

Within the limitations encountered in this literature review, it can be concluded that IARPD is among the viable prosthodontic treatment options for distal extension edentulous areas, which can yield favorable clinical outcomes. Although slight differences were observed between the implant survival rate and mean bone loss in Kennedy’s class I and II, the comparison was not balanced owing to the variations in the study design, number of implants evaluated, and sample size. The stud attachment, especially the ball type, was used most commonly in IARPD treatment, since it is considered to be a simple, economical option, with favorable biological treatment outcomes. The use of different attachment systems overall did not significantly influence implant survival rate and patient satisfaction; however, this aspect and other clinical outcomes should be evaluated statistically, which necessitates the performance of more high-quality studies.

## Data Availability

The data sets generated and analysed during current study are available from the corresponding author upon reasonable request.

## Abbreviations

RPD: Removable partial denture
IARPD: Implant-assisted removable partial denture
ISRPD: Implant-supported removable partial denture
CRPD: Conventional removable partial denture
ISFPD: Implant-supported fixed partial denture
IRPD: Implant-retained partial denture
ERA: Extracoronal resilient attachment
RCT: Randomized controlled trial
